# A systematic outbreak investigation of SARS-CoV-2 transmission clusters in a tertiary academic care center

**DOI:** 10.1186/s13756-023-01242-y

**Published:** 2023-04-21

**Authors:** Matthias von Rotz, Richard Kuehl, Ana Durovic, Sandra Zingg, Anett Apitz, Fanny Wegner, Helena M. B. Seth-Smith, Tim Roloff, Karoline Leuzinger, Hans H. Hirsch, Sabine Kuster, Manuel Battegay, Luigi Mariani, Stefan Schaeren, Stefano Bassetti, Florian Banderet-Uglioni, Adrian Egli, Sarah Tschudin-Sutter

**Affiliations:** 1 Division of Infectious Diseases and Hospital Epidemiology, University Hospital Basel, University of Basel, Petersgraben 4, 4031 Basel, Switzerland; 2 Swiss Tropical and Public Health Institute, Basel, Switzerland; 3 Intensive Care Unit, University Hospital Basel, University of Basel, Basel, Switzerland; 4 Employee Health Service, University Hospital Basel, Basel, Switzerland; 5 Division of Clinical Bacteriology and Mycology, University Hospital Basel, University of Basel, Basel, Switzerland; 6 Applied Microbiology Research, Department of Biomedicine, University of Basel, Basel, Switzerland; 7 Clinical Virology, Laboratory Medicine, University Hospital Basel, University of Basel, Basel, Switzerland; 8 Transplantation and Clinical Virology, Department Biomedicine, University of Basel, Basel, Switzerland; 9 Department of Neurosurgery, University Hospital Basel, University of Basel, Basel, Switzerland; 10 Department of Traumatology and Orthopedics, University Hospital Basel, University of Basel, Basel, Switzerland; 11 Division of Internal Medicine, University Hospital Basel, University of Basel, Basel, Switzerland; 12 Present Address: Institute of Medical Microbiology, University of Zurich, Zurich, Switzerland

**Keywords:** SARS-CoV-2 cluster, COVID-19, Nosocomial outbreaks, Epidemiologic cluster, Whole genome sequencing, Outbreak investigation

## Abstract

**Background:**

We sought to decipher transmission pathways in healthcare-associated infections with severe acute respiratory syndrome coronavirus 2 (SARS-CoV-2) within our hospital by epidemiological work-up and complementary whole genome sequencing (WGS). We report the findings of the four largest epidemiologic clusters of SARS-CoV-2 transmission occurring during the second wave of the pandemic from 11/2020 to 12/2020.

**Methods:**

At the University Hospital Basel, Switzerland, systematic outbreak investigation is initiated at detection of any nosocomial case of SARS-CoV-2 infection, as confirmed by polymerase chain reaction, occurring more than five days after admission. Clusters of nosocomial infections, defined as the detection of at least two positive patients and/or healthcare workers (HCWs) within one week with an epidemiological link, were further investigated by WGS on respective strains.

**Results:**

The four epidemiologic clusters included 40 patients and 60 HCWs. Sequencing data was available for 70% of all involved cases (28 patients and 42 HCWs), confirmed epidemiologically suspected in house transmission in 33 cases (47.1% of sequenced cases) and excluded transmission in the remaining 37 cases (52.9%). Among cases with identical strains, epidemiologic work-up suggested transmission mainly through a ward-based exposure (24/33, 72.7%), more commonly affecting HCWs (16/24, 66.7%) than patients (8/24, 33.3%), followed by transmission between patients (6/33, 18.2%), and among HCWs and patients (3/33, 9.1%, respectively two HCWs and one patient).

**Conclusions:**

Phylogenetic analyses revealed important insights into transmission pathways supporting less than 50% of epidemiologically suspected SARS-CoV-2 transmissions. The remainder of cases most likely reflect community-acquired infection randomly detected by outbreak investigation. Notably, most transmissions occurred between HCWs, possibly indicating lower perception of the risk of infection during contacts among HCWs.

**Supplementary Information:**

The online version contains supplementary material available at 10.1186/s13756-023-01242-y.

## Background

Since the emergence of severe acute respiratory syndrome coronavirus 2 (SARS-CoV-2) in December 2019, healthcare systems have faced the challenge of preventing nosocomial transmission. Initial global shortages of personal protective equipment (PPE), transmission by a- or pre-symptomatic carriers, mild or atypical presentation of disease, high incidence levels in the community affecting both patients and healthcare workers (HCWs) [1], infrastructural conditions, such as multi-bed rooms, poor ventilation systems and overburdened staff, have complicated implementation and maintenance of effective infection prevention and control strategies.

Data exist about the prevalence and associated mortality of nosocomial SARS-CoV-2 infection in hospitalized patients, as well as for the connection between the increase of SARS-CoV-2 infections in HCWs during a general increase of cases in the population. There is, however, limited evidence on the epidemiologic connection between SARS-CoV-2 infections of patients and HCWs correlated with genetic information to describe transmission pathways.

From the beginning of the pandemic until April 2022 we registered 192 potential nosocomial infections at the University Hospital Basel, Switzerland, among 2715 patients with confirmed SARS-CoV-2 infection hospitalized during the same period. Striving to continuously improve our infection prevention and control guidance, we sougth to decipher transmission pathways in all healthcare-associated infections with SARS-CoV-2 by whole genome sequencing (WGS) and thorough epidemiological work-up. We here report the findings of a systematic outbreak investigation of four large clusters of suspected SARS-CoV-2 transmission which occured during the second wave of the pandemic in November and December 2020.

## Methods

### Setting, infection prevention and control measures

The University Hospital Basel is a tertiary care center in Switzerland with more than 40,000 hospital admissions annually. During the study period variants of concern (VOC) were not yet circulating, the mean seven-day incidence of SARS-CoV-2 was 46.9 per 100,000 inhabitants (minimum 29.4, maximum 61.5) in the main catchment area of the hospital (the canton of Basel-Stadt) and the dominant virus strains in Switzerland were B.1.160 and B.1.177. Many differentiable subtypes of these variants were already detectable in Basel.

During this period, restrictions for visitors (visits only allowed after consultation with the ward management for two visitors a day for a maximum of one hour), and universal masking for all visitors, patients and staff were implemented. Patients in multi-bed rooms were advised to eat and drink alone and not at communal tables. Patients diagnosed with SARS-CoV-2 infection were hospitalized on dedicated cohort wards. On these wards, combined contact and droplet precautions were applied and HCWs were required to wear FFP2 masks (or equivalent) and goggles, in addition to gloves and gowns during patient care. Patients with suspected SARS-CoV-2 infection were isolated in multi-bed rooms outside the cohort wards while PCR-results were pending. Isolation precautions comprised wearing surgical masks, gloves and gowns for all direct contacts with the patient or his/her immediate surroundings. The patients’ compliance with surgical mask wearing was prerequisite for isolation in multiple-bed rooms. The patient area was delineated by room dividers or floor markings and dedicated toilets were assigned. A mechanical air filter was placed next to the patient. After confirmation of SARS-CoV-2-infection, patients were reallocated to single-bed rooms or cohorted together with other patients with confirmed SARS-CoV-2 infection. To shorten this time period, antigen testing was performed in addition to PCR for all symptomatic patients. Patients with known exposure to a confirmed coronavirus disease of 2019 (COVID-19) patient were placed in quarantine under combined contact and droplet precautions in a single room for ten days.

### Systematic outbreak investigation

When nosocomial transmission was suspected, all patients hospitalized in the same ward were screened and we recommended a screening for all HCWs working on the respective ward. SARS-CoV-2 was detected by an internally developed reverse transcription quantitative nucleic acid assay [2] and a commercial assay (E-gene; Roche, Rotkreuz, Switzerland) on nasopharyngeal swabs.

#### Whole genome sequencing (WGS) and analysis

Whole genome (Illumina®) sequencing of SARS-CoV-2 was performed on ARTIC amplicons [3] to further analyze clusters of suspected nosocomial transmission. Sequencing was performed on all available isolates with sufficient viral loads for analysis of SARS-CoV-2 ribonucleic acid (RNA) [https://github.com/appliedmicrobiologyresearch/covgap]. All sequences involved in this study have been shared with the Swiss Pathogen Surveillance Platform (www.spsp.ch) and are available on GISAID with the following accession numbers (see Additional file 1).

Sequences were aligned with mafft [4] and a phylogenetic tree was constructed using IQ-TREE2 [5]. The data was analyzed and visualized in R using the packages ape [6], adegenet [7] and ggtree [8]. A transmission event was defined when two sequences were identical (no more than one single-nucleotide polymorphism, SNP).

#### Definition of nosocomial infection, clusters and epidemiological links

In line with the Swiss national surveillance definitions, SARS-CoV-2 infection was defined as nosocomial if diagnosed after day five from hospital admission [9]. A cluster of SARS-CoV-2 infections was defined as the detection of at least two positive patients and/or HCWs within seven days on the same ward (i.e. ward-based exposure) independent of the genomic analysis.

According to the results of the phylogenetic analysis, we further categorized members of the clusters into three subgroups: detection of a genetically identical strain, detection of genetically distinct strain and no sequencing data available. Strain identity was defined as no more than one single-nucleotide polymorphism (SNP) difference between strains by sequence analysis [10]. We further classified each individual’s exposure as “known direct exposure” to another person with known SARS-CoV-2 infection or as “ward-based exposure”. A known direct exposure between two cases was defined as any of the following: (1) between patients: being within the same multi-bed room for a minimum duration of 15 min, and (2) between patients and HCWs: known direct contact during patient care. Exposures between HCWs working on the same ward during overlapping shifts were classified as “ward-based exposure”.

Transmission was considered confirmed when sequencing revealed identity of strains and was ruled out when sequencing revealed distinct strains.

Fisher’s exact test was performed using STATA version 16.1 (StataCorp, College Station, TX) to assess associations between different types of exposures and confirmed transmission. P-values of ≤0.05 were considered significant.

## Results

### Cluster A

**Fig. 1:**
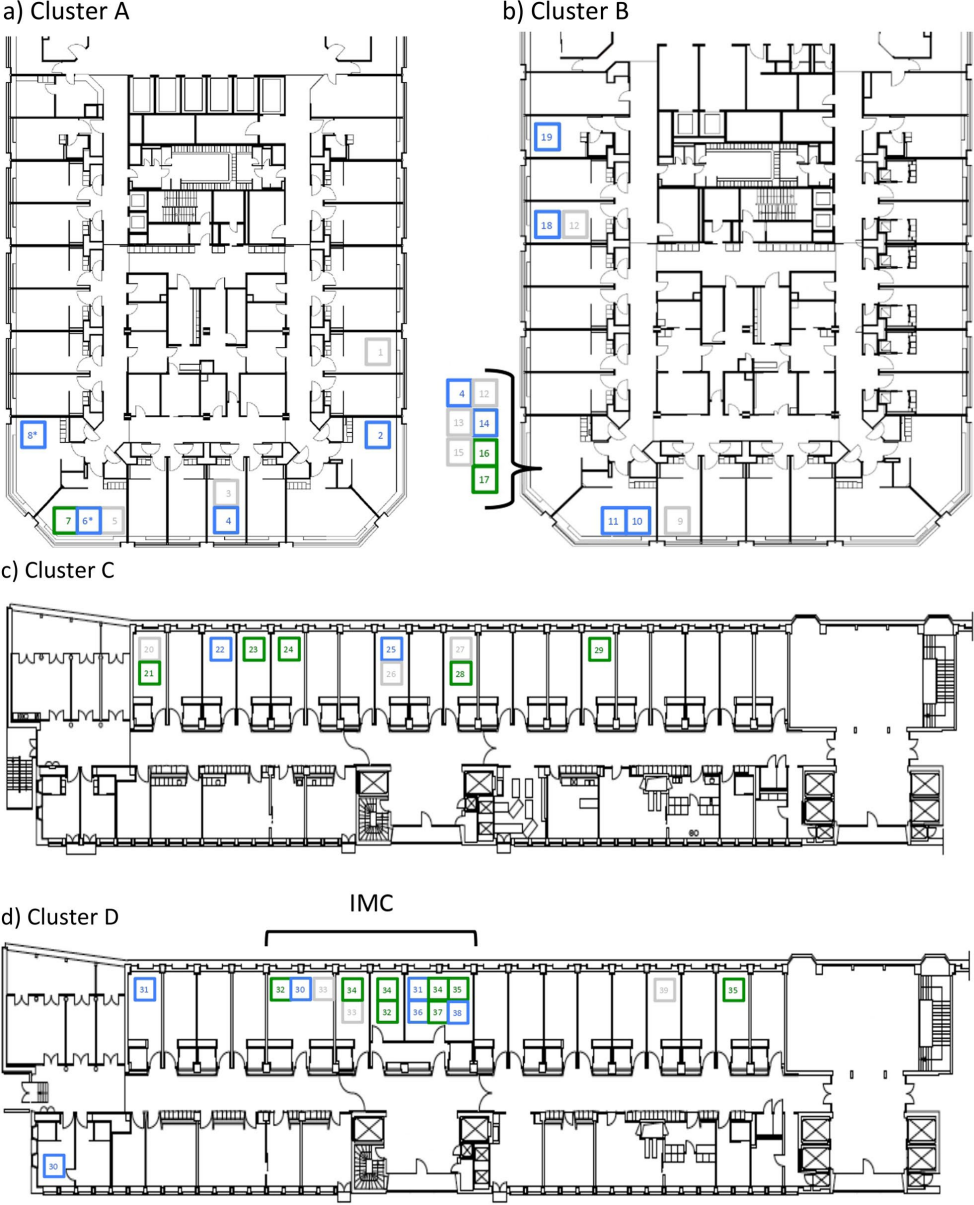
Ward floor plan of Clusters **a**–**d** with colored squares representing SARS-CoV-2 positive patients. Numbers represent individual patients, which may have been hospitalized in different rooms within a ward. The virus-strains of patients represented by a blue square are identical, whereas patients with unique virus-strains are indicated by a green square. Patients with no sequencing data available are represented by grey squares. There were two patients in cluster **a** in adjacent rooms with the same virus-strain, but different to the dominant strain within the cluster (*). The index-patient of cluster **b** (patient number 4) was hospitalized in both wards. Cluster **d** occurred on a ward containing a dedicated intermediate care unit (IMC). In this figure includes 39 rather than 40 patients as one patient-contact occurred on another unit

SARS-CoV-2 infection was confirmed nine days after admission in a patient hospitalized for cardiac failure. Symptoms compatible with COVID-19 were recorded two days earlier. Seven additional patients and five HCWs tested positive for SARS-CoV-2 by subsequent systematic screening and were assigned to cluster A. Infected patients were hospitalized in rooms distributed over the whole ward assigned to three different nursing teams (Fig. 1a). One of the infected patients tested negative at discharge, but was readmitted to the hospital five days later, initiating Cluster B (described below) occurring on a different medical ward.

**Fig. 2:**
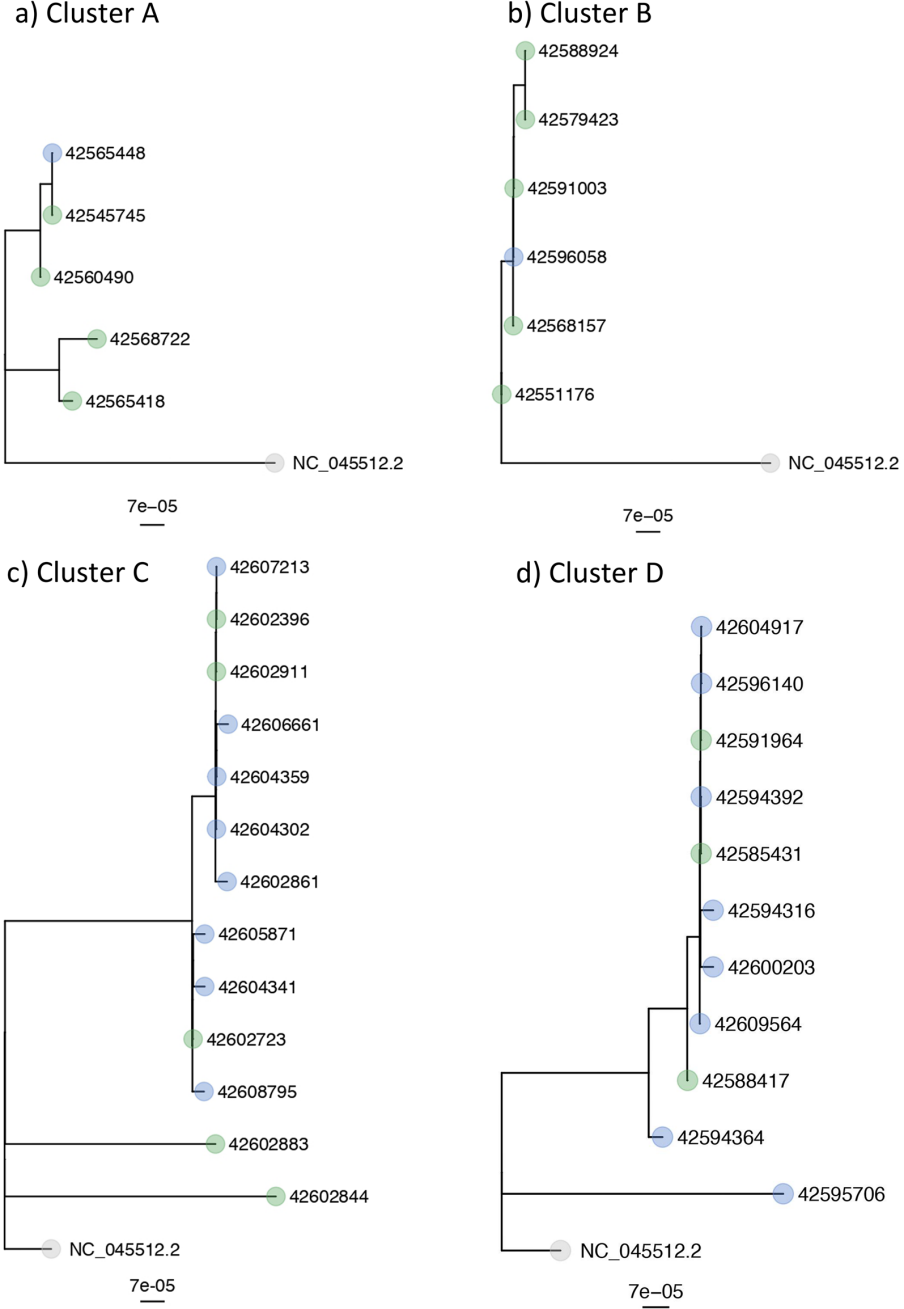
NGS-results of the different clusters in a SNP-tree (single-nucleotide polymorphism tree). The blue dots represent samples from HCWs, whereas patient samples are labelled with green dots. NC_045512.2 (SARS-CoV-2 isolate Wuhan-Hu-1) was used as the reference genome. **a** Cluster A: One patient and three HCWs are missing because the lineage could not be determined due to too low coverage (however, they are unique virus strains or have no genetic association to all other strains within this cluster).** b** Cluster B: Three patients and one HCW are missing because the quality control failed (e.g. low coverage, high percentage of minority variants), they could not be included in the SNP based analysis (however, they have several SNPs difference to all other strains within this cluster)** c** Cluster C: Two patients and six HCWs are missing because the quality control failed (e.g. low coverage, high percentage of minority variants), they could not be included in the SNP based analysis (one additional HCW in the cluster, otherwise different strains).** d** Cluster D: Five patients and 14 HCWs are missing because the quality control failed (e.g. low coverage, high percentage of minority variants), they could not be included in the SNP based analysis

WGS was successful for five of the eight patients and four of the five HCWs (Table 1). Based on phylogenetic analyses (Fig. 2a), two different SARS-CoV-2 strains were transmitted (both refer to lineage B.1.160) on this ward: one among two patients and one HCW, each with no known direct exposure and the other between two patients hospitalized in adjacent rooms. Unique virus strains were identified in the remaining patient and three HCWs. As exposure was classified as ward-based for all confirmed transmission events (Table 1), transmission most likely occurred via HCWs possibly acting as vectors, as patients were less likely to have had contact between each other beyond their individual rooms. Estimating timing based on symptom onset, patient to HCW transmission seems likely to have occurred in one of four of the affected HCWs.

**Table 1:** Characteristics of the different clusters

		Cluster A (internal medicine ward)	Cluster B (internal medicine ward)	Cluster C (surgical ward)	Cluster D (surgical ward)
** People affected, n (patients /HCW)**		13 (8/5)	16 (12/4)	27 (10/17)	44 (10/34)
WGS available, n (%) Patients, n (%) HCWs, n (%)		9 (69.2%) 5/8 (62.5%) 4/5 (80.0%)	10 (62.5%) 8/12 (66.7%) 2/4 (50.0%)	21 (77.8%) 7/10 (70.0%) 14/17 (82.4%)	30 (68.2%) 8/10 (80.0%) 22/34 (64.7%)
**Transmission among people with available sequencing data**					
Confirmed, n (%)		5* (55.6%)	6 (60.0%)	8 (38.1%)	14 (46.7%)
Ruled out, n (%)		4 (44.4%)	4 (40.0%)	13 (61.9%)	16 (53.3%)
**People with genetically identical strains, n (%)** Patients /HCW		5* (38.5%) 4/1	**6 (37.5%)** 5/1	**8 (29.6%)** 2/6	**14 (31.8%)** 4/10
Direct exposure					
Exposure between patients		0	3	0	3
Exposure between patient and HCW		0	1 (HCW)	1 (P), 1 (HCW)	0
Ward-based exposure		4 (P), 1 (HCW)	2 (P)	1 (P), 5 (HCW)	1 (P), 10 (HCW)
**People with genetically distinct strains, n (%)** Patients /HCW		**4 (30.8%)** 1/3	**4 (25.0%)** 3/1	**13 (48.1%)** 5/8	**16 (36.4%)** 4/12
Direct exposure					
Exposure between patients		1	3	0	3
Exposure between patient and HCW		1 (HCW)	1 (HCW)	3 (HCW)	2 (HCW)
Ward-based exposure		2 (HCW)	0	5 (P), 5 (HCW)	1 (P), 10 (HCW)
**People with missing sequencing data, n (%)** Patients /HCW		**4 (30.8%)** 3/1	**6 (37.5%)** 4/2	**6 (22.2%)** 3/3	**14 (31.8%)** 2/12
Direct exposure					
Exposure between patients		2	2	3	0
Exposure between patient and HCW		0	0	0	3 (HCW)
Ward-based exposure		1 (P), 1 (HCW)	2 (P), 2 (HCW)	3 (HCW)	2 (P), 9 (HCW)
**Main virus lineage**		B.1.160	B.1.160	B.1.177	B.1.177
**Department screenings, n (Patients /HCW)**		2/2	3/0	2/2	3/2
**Clinical course (Patients) **					
Asymptomatic		2	0	6	2
Symptomatic		2	9	3	6
Deceased		4	2	0	2
Unknown		0	1	1	0

*Consists of two different clusters (two and three identical virus-strains, respectively)

*HCW * Health-care worker,* WGS*  Whole genome sequencing,* P*  Patient

### Cluster B

The index-patient of this cluster occurring on an internal medicine ward was exposed to cluster A during the previous hospital stay and was readmitted five days after discharge due to suspected COVID-19. Isolation measures were implemented in a multi-bed room while PCR results were pending. After confirmation of SARS-CoV-2 infection, this patient was moved to the dedicated cohort unit. Six days later, a patient hospitalized in the same multi-bed room for six hours developed symptoms and was diagnosed with COVID-19. Another patient hospitalized in the same multi-bed room (directly exposed to the index patient for 30 min) tested positive for SARS-CoV-2 six days later (no sequencing data available). Yet, four days later, his contact patient tested positive too (genetically identical virus strain as the index patient). In this cluster, nine more patients were infected with SARS-CoV-2 (five direct patient to patient exposures), as well as four HCWs within the next 18 days. Patient room distribution is shown in Fig. 1b.

Phylogenetic analysis confirmed the suspected initiation of this cluster by the index patient. Overall, four HCWs in this cluster were infected with SARS-CoV-2 (two of them without direct exposure to the affected patients).

Sequencing data was available for eight patients and two HCWs (Fig. 2b). Transmission from a patient to one HCW is likely to have occurred (diagnosed five days later), as the HCW reported direct exposure and sequencing data confirmed the detection of an identical strain

### Cluster C

The index case of this cluster was a patient hospitalized on a surgical ward who tested positive for SARS-CoV-2 on day 12 after admission for suspected intestinal perforation. Transmission to the direct contact patient sharing the same room during five days was confirmed one day later. A ward screening for SARS-CoV-2 revealed an additional six infected patients, one with a persistent positive SARS-CoV-2 test after an infection one month prior. Two further contact patients became positive for SARS-CoV-2 three and six days later respectively. Patient room distribution is shown in Fig. 1c. At cluster onset, four positive HCWs were identified on this ward. Two subsequent systematic screenings of HCWs revealed 13 additional infections within the next eight days.

Sequencing was successful for seven patients and 14 HCW (Table 1). Phylogenetic analysis (Fig. 2c) confirmed presence of the same strain in eight, yet revealed different strains in 13 cases.

### Cluster D

Cluster D occurred on a surgical ward with an associated intermediate care unit for neurosurgery patients. The index patient was screened positive for SARS-CoV-2 on day 11 of the hospital stay. Six days later a second patient tested SARS-CoV-2 positive eight days after admission. As a HCW on the same ward, caring for both patients, tested positive at the same time, subsequent screening of patients and HCWs of the affected ward was initiated. Overall, 34 HCWs and 10 patients tested positive within a time frame of 18 days. Eight of the ten affected patients were transiently hospitalized in the intermediate care unit. As infection onset occurred concurrently in five of the affected patients, transmission is likely to have occurred via an infected HCW. In addition, three patients hospitalized in the same room as infected patients (prior to detection) were infected, too.

Factors potentially facilitating transmission between HCWs and patients within the intermediate care settings are likely the need for prolonged care with close contact. Patient room distribution is shown in Fig. 1d. We assume contacts between the HCWs being the main driver for ongoing transmission within this cluster mainly affecting employees.

Phylogenetic analysis confirmed presence of the same strain in four patients and ten HCWs (Fig. 2d) and excluded transmission in four patients and 12 HCWs despite two HCWs reporting direct exposure to infected patients. Sequencing data was not obtainable for two patients and 12 HCWs.

### General considerations

Overall, sequencing data was available for 42 HCWs and 28 patients (70% of all people involved in one of the four clusters). It confirmed epidemiologically suspected transmission events in 47.1% (53.6% of all involved patients, and 42.9% of all involved HCWs with available sequencing data) and ruled out transmission in 52.9%. Among all direct exposure events, sequencing data confirmed transmission in 39.1% (9/23) and ruled out transmission in 60.9% (14/23). No type of exposure was associated with confirmed transmission (overall p-value = 0.447, overall p-value for direct exposure events* p* = 1.000). Most confirmed transmission events resulting from direct exposure (n = 9) occurred during contacts between patients (6/9, 66.7%), followed by contacts between HCWs and patients (3/9, 33.3%). The majority of confirmed transmission resulted from a ward-based exposure (24/33, 72.7%). Among HCWs reporting a direct exposure to a patient, transmission was confirmed in 22.2% (2/9).

Among all HCWs with available sequencing data, patient contacts resulting in confirmed transmission accounted for 7.2% (3/42) of all infections. The major part of virus-strains of HCW were unique (52.4%, 22/42), while a ward-based exposure (and contacts between HCWs) resulted in confirmed transmission in 40.5% (17/42).

Among all patients involved in one of these four clusters, disease was asymptomatic in 27.5% (11/40) and symptomatic for 67.5% (27/40) (Table 1). Eight patients died (20%), COVID-19 contributing to this fatal outcome in addition to underlying diseases.

## Discussion

Phylogenetic analyses revealed important insights into transmission pathways supporting only 47% of epidemiologically suspected SARS-CoV-2 transmissions within the context of nosocomial outbreak investigation. The remainder of cases most likely reflect community-acquired infection randomly detected by broad screening efforts. These results indicate that calculations of attack rates, not taking sequencing data into account, may result in an overestimation of the transmission risk allocated to specific hospital exposures. Notably, most confirmed transmissions occurred between HCWs, possibly indicating lower perception of the risk of infection among colleagues working together.

Our results indicate that late recognition of infected patients was the main starting point for these four clusters of nosocomial infection as reported by other outbreak investigations [11]. Importantly, complementary analyses of the sequencing data revealed a far more complex picture supporting multiple introductions of distinct strains, some entertaining different sub-clusters among patients and/or HCWs. This is meaningful, since epidemiological work-up would have concluded that all clusters were attributable to few unique sources maintaining onward transmission to patients and HCWs. This finding points to the need to pursue multi-facetted interventions targeting patients and HCWs to break nosocomial transmission chains. Such interventions include strategies for early detection of a- or pre-symptomatic infections but point to the need of enhanced universal precautions, such as mask-wearing and distancing, especially during phases with high-levels of community transmission.

Healthcare workers are at increased occupational risk of acquiring SARS-CoV-2 [12, 13]. This risk has been mainly considered to be resulting from exposures to infectious patients [14, 15]. Our findings suggest that community-acquired infection is the most common route of infection among HCWs, supported by the majority of HCWs being infected with genetically distinct SARS-CoV-2 strains. The importance of community-acquired infection among HCWs has been previously suggested [16–18] and the rate of asymptomatic infection among HCWs has been shown to more likely reflect general community transmission than in-hospital exposure [19]. Our interpretation is supported by our findings that only 7% of all sequenced strains collected from HCWs in our report were shared between HCWs and patients, while 93% of HCW-strains were either unique (52%) or shared between HCWs (41%, ward-based exposure). Among HCWs reporting a direct exposure to a patient, transmission was ruled out in 78%. This finding suggests that our infection prevention and control measures were suitable to avoid onward transmission from patients by asymptomatic or pre-symptomatic carriers in the majority of cases. Among occupational exposures resulting in confirmed transmission, direct contacts between HCWs were the most common route of transmission. The importance of SARS-CoV-2 transmission between HCWs is supported by previous outbreak investigations involving staff working across different care homes in London [20] and from a large UK NHS Trust [21]. During our outbreak investigations, we commonly identified insufficient social distancing between HCWs during coffee- and meal breaks as a relevant source entertaining transmission between HCWs, especially under tighter space conditions. Further social interactions, such as in common smoking areas, car pools or private contacts may further contribute. A qualitative study identified high-risk interactions between HCWs during handoffs of care at shift changes and patient rounds, when HCWs gathered regularly in close proximity for at least 15 min. Identified barriers included spacing and availability of computers, the need to communicate confidential patient information, and the desire to maintain relationships at work [22].

It is, however, noteworthy, that HCWs commonly reported patients as the most likely source of infection, suggesting a flawed conception of perceived and actual risk. Among critical care staff, the peak onset of COVID-19 symptoms has been shown to occur two weeks before the peak in COVID-19 patient admissions with staff working in multiple hospital departments, thus exposed to more diverse co-worker encounters or with symptomatic household contacts more likely being infected [23].

Secondary attack rates of 19% have been previously reported for patients sharing the same room with an unrecognized infected patient [24]. It is noteworthy, that direct exposure between patients (defined as sharing the same multi-bedroom for a minimum duration of 15 min), resulted in confirmed transmission of SARS-CoV-2 in only 46% of patients testing positive after such exposure, given that most patients were not able to wear masks continuously during their stay in the room and were often exposed to an unrecognized infectious patient for several hours. In contrast, mostly shorter exposures as during HCW-patient interactions or contacts between HCWs resulted in similar transmission rates, suggesting that close contact as encountered during patient care or joint meal breaks result in a higher risk of transmission over time. Close contact to an infected HCW during patient care has been previously reported as an important route of transmission for nosocomial infections of patients [25].

Our findings have several limitations. First, sequencing data was not obtainable for all patients and HCWs involved in the reported infection-clusters. The proportion of successful sequencing in our study (70%) was nevertheless similar to a previous study investigating nosocomial infections in the UK (72%) [11]. Second, data on direct exposures may be flawed by recall-bias and short-term changes to working plans. As these outbreak investigations were performed in November and December 2020, our findings may not be generalizable to in-hospital transmission of other SARS-CoV-2 variants or populations with higher levels of immunity (acquired by infection and/or vaccination, the latter not being available yet for patients and HCWs at that time in Switzerland).

## Conclusions

WGS analyses revealed important insights into transmission pathways supporting only 50% of epidemiologically suspected SARS-CoV-2 transmissions. The remainder of cases most likely reflects community-acquired infection randomly detected by outbreak investigation. Notably, most transmissions occurred between HCWs, possibly indicating lower perception of the risk of infection among HCWs.

## Supplementary Information

**Additional file 1.** List of all sequences involved in this study. These sequences have been shared with the Swiss Pathogen Surveillance Platform (www.spsp.ch) and are available on GISAID with these accession numbers.

## Data Availability

The dataset generated and analyzed during the current study is not publicly available due to individual privacy possibly being compromised but is available from the corresponding author on reasonable request.

## Abbreviations

COVID-19: Coronavirus disease of 2019
EKNZ: Ethics Commission of Northwestern and Central Switzerland
FFP2: Filtering face piece 2
HCWs: Healthcare workers
IMC: Intermediate care unit
P: Patient
PCR: Polymerase chain reaction
PPE: Personal protective equipment
RNA: Ribonucleic acid
SARS-CoV-2: Severe acute respiratory syndrome coronavirus 2
SNP: Single-nucleotide polymorphism
UK NHS Trust: United Kingdom National Health Services Trust
VOC: Variants of concern
WGS: Whole genome sequencing
