# Supplementary material for: A systematic outbreak investigation of SARS-CoV-2 transmission clusters in a tertiary academic care center
Source: Antimicrob Resist Infect Control. 2023 Apr 21;12:38. doi: 10.1186/s13756-023-01242-y (PMC10119817; doi:10.1186/s13756-023-01242-y)
Supplement: Supplementary file 1 — Additional file 1. List of all sequences involved in this study. These sequences have been shared with the Swiss Pathogen Surveillance Platform (www.spsp.ch) and are available on GISAID with these accession numbers. [file 13756_2023_1242_MOESM1_ESM.docx]

Supplementary table:

| **ID** | **Virus_strain** | **Accession_ID** |
| --- | --- | --- |
| 42545745 | hCoV-19/Switzerland/BS-UHB-42545745/2020 | EPI_ISL_931361 |
| 42560490 | hCoV-19/Switzerland/BL-UHB-42560490/2020 | EPI_ISL_931365 |
| 42565448 | hCoV-19/Switzerland/BS-UHB-42565448/2020 | EPI_ISL_931368 |
| 42565418 | hCoV-19/Switzerland/BL-UHB-42565418/2020 | EPI_ISL_931367 |
| 42568722 | hCoV-19/Switzerland/AG-UHB-42568722/2020 | EPI_ISL_931370 |
| 42551176 | hCoV-19/Switzerland/BS-UHB-42551176/2020 | EPI_ISL_931363 |
| 42568157 | hCoV-19/Switzerland/BS-UHB-42568157/2020 | EPI_ISL_931369 |
| 42579423 | hCoV-19/Switzerland/BL-UHB-42579423/2020 | EPI_ISL_931392 |
| 42591003 | hCoV-19/Switzerland/BL-UHB-42591003/2020 | EPI_ISL_1388864 |
| 42596058 | hCoV-19/Switzerland/BS-UHB-42596058/2020 | EPI_ISL_1388685 |
| 42588924 | hCoV-19/Switzerland/BS-UHB-42588924/2021 | EPI_ISL_1747647 |
| 42608795 | hCoV-19/Switzerland/BL-UHB-42608795/2020 | EPI_ISL_1388860 |
| 42604341 | hCoV-19/Switzerland/BS-UHB-42604341/2020 | EPI_ISL_1388868 |
| 42604359 | hCoV-19/Switzerland/BS-UHB-42604359/2020 | EPI_ISL_1388869 |
| 42607213 | hCoV-19/Switzerland/BL-UHB-42607213/2020 | EPI_ISL_1388942 |
| 42606661 | hCoV-19/Switzerland/BS-UHB-42606661/2020 | EPI_ISL_1388941 |
| 42602861 | hCoV-19/Switzerland/BS-UHB-42602861/2020 | EPI_ISL_1388935 |
| 42602844 | hCoV-19/Switzerland/JU-UHB-42602844/2020 | EPI_ISL_1388934 |
| 42605871 | hCoV-19/Switzerland/BL-UHB-42605871/2020 | EPI_ISL_1388939 |
| 42602883 | hCoV-19/Switzerland/BS-UHB-42602883/2020 | EPI_ISL_1388936 |
| 42602723 | hCoV-19/Switzerland/BL-UHB-42602723/2020 | EPI_ISL_1388933 |
| 42602396 | hCoV-19/Switzerland/AG-UHB-42602396/2020 | EPI_ISL_1388932 |
| 42602911 | hCoV-19/Switzerland/BL-UHB-42602911/2020 | EPI_ISL_1388937 |
| 42604302 | hCoV-19/Switzerland/SO-UHB-42604302/2020 | EPI_ISL_1389002 |
| 42588417 | hCoV-19/Switzerland/BL-UHB-42588417/2020 | EPI_ISL_931393 |
| 42591964 | hCoV-19/Switzerland/BL-UHB-42591964/2020 | EPI_ISL_931394 |
| 42594364 | hCoV-19/Switzerland/BL-UHB-42594364/2020 | EPI_ISL_1388666 |
| 42585431 | hCoV-19/Switzerland/GR-UHB-42585431/2020 | EPI_ISL_1388900 |
| 42595706 | hCoV-19/Switzerland/BL-UHB-42595706/2020 | EPI_ISL_1388201 |
| 42594392 | hCoV-19/Switzerland/BS-UHB-42594392/2020 | EPI_ISL_1388926 |
| 42596140 | hCoV-19/Switzerland/BS-UHB-42596140/2020 | EPI_ISL_1388244 |
| 42600203 | hCoV-19/Switzerland/BL-UHB-42600203/2020 | EPI_ISL_1388930 |
| 42609564 | hCoV-19/Switzerland/BS-UHB-42609564/2020 | EPI_ISL_1388283 |
| 42604917 | hCoV-19/Switzerland/BL-UHB-42604917/2020 | EPI_ISL_1389008 |
| 42594316 | hCoV-19/Switzerland/SO-UHB-42594316/2020 | EPI_ISL_1388275 |
